# Enhancing learning in a perceptual-cognitive training paradigm using EEG-neurofeedback

**DOI:** 10.1038/s41598-021-83456-x

**Published:** 2021-02-18

**Authors:** Brendan Parsons, Jocelyn Faubert

**Affiliations:** 1 Department of Psychology, Université de Montréal, Montreal, Canada; 2 Faubert Lab, School of Optometry, Université de Montréal, Montreal, Canada

**Keywords:** Neuroscience, Psychology

## Abstract

This paper provides the framework and supporting evidence for a highly efficient closed-loop paradigm that modifies a classic learning scenario using real-time brain activity in order to improve learning performance in a perceptual-cognitive training paradigm known as 3-dimensional multiple object tracking, or 3D-MOT. Results demonstrate that, over 10 sessions, when manipulating this novel task by using real-time brain signals, speed and degree of learning can be substantially improved compared with a classic learning system or an active sham-control group. Superior performance persists even once the feedback signal is removed, which suggests that the effects of enhanced training are consolidated and do not rely on continued feedback. This type of learning paradigm could contribute to overcoming one of the fundamental limitations of neurofeedback and other cognitive enhancement techniques, a lack of observable transfer effects, by utilizing a method that can be directly integrated into the context in which improved performance is sought.

## Introduction

Of the numerous domains in which neuroscience is being widely applied, learning and cognitive enhancement figure prominently in both scientific research and popular culture. While there is a burgeoning interest in these techniques and technologies, one major issue is still to be addressed. Evidence for *transfer*, the replication or application of an enhanced ability in a context other than that of the training paradigm, is largely lacking^1^.

Cognitive enhancement paradigms typically target given function or set of cognitive abilities, train them in a given paradigm, and then use standardized tests and outcome measures to demonstrate cognitive enhancement and transfer. While cognitive enhancement appears attainable in a number of domains, evidence of transfer remains severely limited^1^. This novel research proposes integrating EEG-neurofeedback directly into an existing learning paradigm in order to demonstrate that it is possible to overcome the weak transfer effects observed in existing cognitive enhancement and neurofeedback literature^2^ by applying enhancement directly into the target context.

Banich and Compton define *skill learning* as “the acquisition—usually gradually and incrementally through repetition—of motor, perceptual, or cognitive operations or procedures that aid performance.”^3^ Here, we are especially interested in the perceptual and cognitive operations. Since all learning is the result of neuroplasticity, an updated brain-based approach to learning, using all of the technological advancements of the past decades, is fundamentally needed.

**Figure 1:**
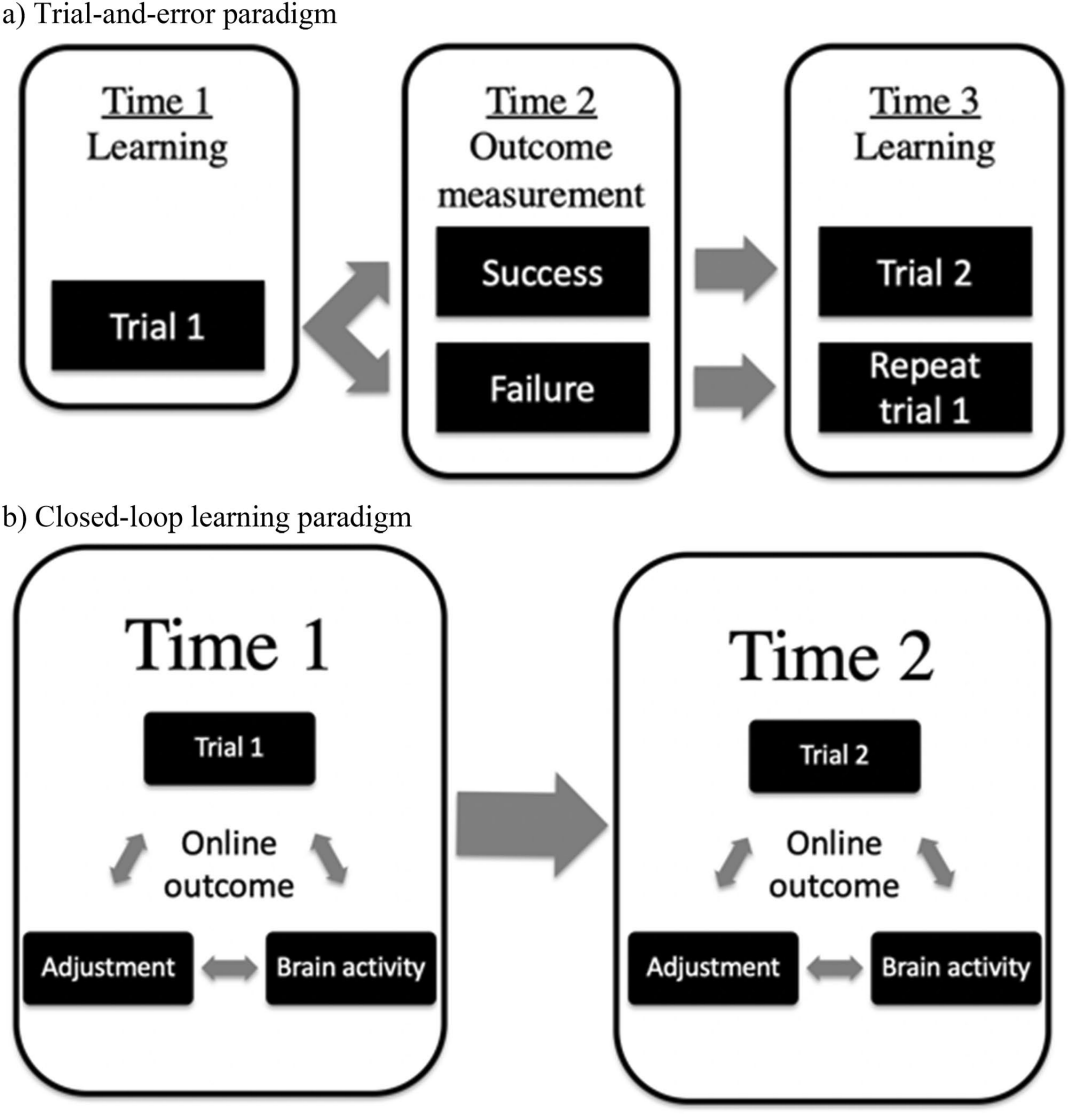
Illustrations of traditional versus looped learning paradigms. (**a**) In a traditional trial-and-error learning paradigm, the entirety of time 1 is spent learning. A separate instance, time 2, is dedicated to measuring the outcome of learning, and can either result in success or failure. The next step, time 3, is either dedicated to learning trial 2 (if the result of outcome measurement was success) or repeating trial 1 (ideally with an adjustment, and in the case of failure at time 2). (**b**) In a closed-loop learning paradigm, time 1 is spent learning, but includes online error-detection based on brain activity and real-time adjustment, which modifies the manner in which learning occurs to ensure successful learning before moving onto trial 2. Arrows are bidirectional since learning and adjustment has an effect on the brain, which in turn drives the learning process and necessary adjustment.

In developed parts of the world, a large part of skill learning comes from academic education. The developed world acknowledges some shortfalls of these systems, however, still tout them as the best^4^. Yet, from preschools to the most sophisticated institutions of higher learning, we are still essentially using *trial and error learning*^5^. The base formula for this system is: spend time and energy trying to learn something, and then apply a post-hoc test to see if learning has occurred; thus providing *feedback*. If learning has occurred, move on to the next subject. If it does not, repeat (hopefully with a variation based on experience from the failed trial) and test again. This traditional type of learning paradigm is presented in Fig. 1a. The so-called ‘solution’ of simple repetition, though, appears to fall short in academic contexts^6^.

While we continue to proceed with trial and error learning, the manner in which we teach has at least somewhat evolved over time, in large part due to the great work of Jean Piaget, Leonard Vygotsky, Jerome Bruner and many others. Their work led to the fundamental tenant that drives our current learning systems: *the zone of proximal development*^7^. This involves maintaining the difficulty of a task within a certain degree of difficulty, or simply put: in order to maximize learning, a task should never be too hard or too easy^7^.

Clow^8^ articulates a framework for successful learning cycle: “successful learning analytics work as four linked steps: learners (1) generating data (2) that is used to produce metrics, analytics or visualisations (3). The key step is ‘closing the loop’ by feeding back this product to learners through one or more interventions (4).” The same author proposes improving learning by “speeding up or shortening the cycle so feedback happens more quickly” ^8^.

A brain-based approach aims to optimize a learning paradigm based on the leaner’s *real-time* ability to learn by providing feedback based on the moment-to-moment changes in the brain. A real-time indicator of performance is provided, allowing consistent real-time feedback instead of waiting for a post-hoc test. What we are referring to is a *closed-loop learning paradigm*. In other words, take the information that a person is perceiving, processing, understanding and encoding into their brain in a given environment at a given moment in time, and feed it to the brain optimally, using the brain’s own performance to adjust the flow of information and the task as appropriate. Figure 1b provides a graphic representation of this paradigm.

To further illustrate this concept, let’s take an example in which learning can occur and simplify it. A significant amount of learning can involve *reading*, so let’s start there. First, you’d have to measure the neural activity in the part of the brain (Wernicke’s area) responsible for the reading task^9^, and provide the learning stimulus (the next word or sentence) to a person only when this area of the brain is functioning optimally and thus receptive to the information.

While this is great in theory, it is an oversimplified idea that rests on a number of assumptions. First, we are assuming that Wernicke’s area is solely responsible for reading comprehension, which is not the case^10^. It also assumes that we can define and measure what the optimal functioning of this part of the brain is, and although this is theoretically possible, it is quite complex^11^. Finally, it assumes that the suggested manipulation is going to maintain the task within the zone of proximal development, and thus facilitate a task that is otherwise too difficult, and load a task that is otherwise too easy.

With all of these assumptions, one would think that a rather complex framework for a closed-loop paradigm is needed, but thanks to technological advancements and mounting evidence from various domains of neuroscience, this type of paradigm is possible today.

Dating back to the 1970s, brain-computer interfaces have been used to measure real-time brain activity and feed the signal back to the person^12^ in order to modify brain function^13^ and structure^14^. Called *neurofeedback* (NFB), this technique has been employed in order to diminish symptoms of various cognitive difficulties and disorders (ADHD^12^, epilepsy^15^, depression and anxiety^16^ and others^17^), and enhance cognitive abilities (attention^18^, working memory^17^, music performance^19^ and others^20^) in cognitively healthy individuals. It is typically applied as a conditioning procedure involving the modulation of visual (animations) and auditory stimuli (music) ^16^.

One type of brain activity often targeted by neurofeedback protocols, alpha, is typically defined as neural oscillations at a speed between 8 and 12 or 13 Hz^21^. *Peak Alpha Frequency*, or PAF, “corresponds to the discrete frequency with the highest magnitude within the alpha range” ^22^. Although EEG measures cortical activity, in the case of the upper-band alpha activity examined here, the source of this activity is primarily believed to be the thalamus and thalamo-cortical loops^23^.

The thalamus is often referred to as a high-order relay station; an important router tasked with dispatching incoming information and coordinating relevant parts of the brain for more complex work^24^. Succinctly, the thalamus sends the relevant information to the cortex, the executor of the task. The cortex then responds to the thalamus indicating whether or not it has achieved the work it was asked to do, giving feedback on its performance. This back-and-forth circuitry between the thalamus and the cortex is referred to as a *thalamo-cortical loop*^25^. In adults, the speed of these thalamo-cortical loops is approximately 10 Hz and varies faster and slower based on cognitive performance; hence the measure of the PAF^26^. A significant correlation between the speed of these oscillations and task performance has been shown: a faster PAF means better performance on a wide variety of tasks^27^.

This leads to the idea that while the thalamus can be interpreted as the “router” of cortical activity, the PAF is then the speed at which the “router” is able to dispatch relevant signals throughout the brain and receive a response back. By this understanding, a faster PAF means that the thalamus (router) is dispatching information to the cortex (effector), and providing a return signal (feedback), more efficiently. One could even choose to think of it as another case of trial and error; the thalamus sends a task to the cortex (trial) and the cortex communicates back the result (success or error). PAF then, in this paradigm, serves to indicate an indirect measure of learning success or failure by signalling proper workload (success; faster PAF) or inefficient functioning (error; slower PAF). Essentially then, the result is a measure of trial-and-error learning in near real-time based on an objective, quantifiable brain measure.

Subset of neurofeedback protocols have specifically targeted the higher frequencies of the alpha band or set out to increase the dominant or peak alpha frequency. Notably used to enhance processing speed and executive function in healthy elderly populations^28^, memory in aging populations with mild cognitive impairment^29^, recovery from spatial neglect in stroke patients^30^, but also in healthy individuals for the enhancement of cognitive performance^31^, this type of neurofeedback has also been demonstrated to augment abilities in a mental rotation task^20^, short term memory^32^, and working memory^33^.

In these “classic” paradigms, the target parameters provide feedback by modulating a neutral, salient stimulus, and conditioning is driven by the basic principles of reward/punishnment^2^. This is a major difference with the approach described herein. Here, we propose the use of neurofeedback within a specific, applied learning paradigm to create a closed-loop learning system that uses the brain’s real-time activity to manipulate a specific task in order to enhance and optimize learning.

Other types of neurofeedback paradigms have been proposed, often by simultaneously combining classic neurofeedback with a cognitive task (for example, see Hosseini and colleagues^34^) while others have proposed creating neurofeedback-driven video-games to make training more enjoyable^35^. One very limited pilot study integrated a neurofeedback-modulated variable into a “shooter” style video game, and demonstrated changes in the peak alpha frequency in the three subjects trained^36^. While this latter work is closest to the proposed paradigm, a distinct difference in this work is that neurofeedback was integrated into a pre-existing learning paradigm (3D-MOT; see “Method” section below) that has been applied in various contexts, with measurable transfer effects. In the famework of the learning model set out by Clow^8^, this significant difference makes the currently proposed paradigm distinct, in that it is an entirely closed-loop learning system.

There are a few steps required to set up a closed-loop system. Specifically, one must (1) identify the task, (2) identify the relevant brain regions and networks involved, (3) define what the optimal functioning of these regions and network is and (4) define and validate the manipulation of the task that will enhance learning.

That is exactly what we have set out to do: demonstrate that in the case of a novel learning paradigm, by targeting brain functioning even with relatively low specificity, a closed-loop system enhances learning when compared with a traditional paradigm, an active-sham control group and a non-active control group.

## Method

### The task

For the purposes of this investigation, in order to respect a typical learning paradigm, a trial-and-error task was chosen. The task used here is a perceptual cognitive paradigm known as 3-dimensional multiple object tracking, or 3D-MOT^37–40^. The 3D-MOT task involves tracking multiple target spheres amongst distractor spheres through 3-dimensional cube. Four targets are used as research has shown that most people, notably healthy adults, can generally track four elements in such a context^37^.

Parsons and colleagues^40^ provide the following breakdown of a 3D-MOT trial: “During the first phase of each trial, all 8 spheres appear in yellow and are stationary. Next, the 4 target spheres that the trainee must track appear in red for 2 s, before switching back to yellow. The spheres begin movement and tracking then occurs over a period of 8 s. All 8 spheres move along a linear path through the cube; should any sphere encounter an obstacle (either a wall or another sphere) it bounces off that obstacle and continues along its new linear trajectory. At the end of this phase, each sphere is identified with a number and the trainee is asked to verbally state their responses” ^40^.

The 3D-MOT task lends itself well to this research, as it follows the same standard “trial and error” approach as traditional learning paradigms. If all 4 targets are correctly identified the speed of the subsequent trial (the task difficulty) increases. If an incorrect response is given, the speed of the subsequent trial decreases, decreasing task difficulty. The speed changes are based on an adaptive staircase with wider initial variances followed by progressively smaller changes. This ensures that the learner quickly enters their zone of proximal development. At the end of a series of 20 trials, a final speed threshold score is given which reflects the maximum speed at which a subject’s performance is adequate (identification of all 4 correct targets). A subject’s session score comprises the average threshold score of 3 series of 20 trials.

3D-MOT training has been demonstrated to enhance attention, working memory and visual information processing speed in healthy participants^40^, attention in those with neurodevelopmental deficits^41^, on-field performance in athletes^42^, and memory^43^ and biological motion processing in healthy aging participants^44^.

### Neural correlates

Being a visuospatial attentive tracking task, the neural network colloquially known as the *where* stream of visual processing is heavily involved^45^. The dorsal (*where)* stream is a complex visual network involved in locating and tracking objects through space, as opposed to the ventral (*what)* stream responsible for object identification^46^. A number of cerebral regions are involved in the *where* stream, most of them located around the dorsal posterior cortex^47^. A critical point, the precuneus (which corresponds to 10–20 location Pz)^47^, is easily measured using electroencephalographic (EEG) sensors placed on the scalp. EEG sensors passively measure the brain’s electric activity^27^. Due to its non-invasiveness, ease of use, low cost and temporal specificity, EEG was selected for the purposes of this study.

### Optimal functioning

Thus far, progress into designing a closed-loop paradigm has been relatively simple. When it comes to defining the optimal functioning of the brain during the 3D-MOT task, or more specifically of the critical parts of the brain involved in the task (referenced by the electrode site Pz above), the precuneus and medial parietal cortex, there are a considerable number of variables to consider. These include the specifics of the task, the neural networks involved, and the individual performing the task.

Optimal functioning within a learning paradigm requires a targeted workload that falls within or slightly above the domain of current competence^7^; thus defining and measuring workload is essential. *Workload* can be defined as “the amount of mental resources that are used to execute a specific task, also known as working memory load.”^48^ This model of workload, developed and utilized by Gerjets et al. ^49^, among others, has been applied in a multitude of learning tasks with and without a brain-computer interface (BCI) ^50^.

Many EEG markers for workload have been proposed, from the relatively simple (for example: frequency band amplitude measures^51^ and ERPs^52^) to the complex (for example: adaptive deep-learning models^53^). For the purposes of this study, a specific element of EEG activity, a relatively general measure of cognitive performance known as *Peak Alpha Frequency* (PAF) was selected as the target variable. The relationship between PAF and cognitive performance extends back to the late 1930s and early 1940s, when Knott^54^ and Hadley^55^ reported changes in the dominant alpha rhythm using only visual (“eyeball”) inspection. More advanced technologies as early as the 1980s allowed for more precise power spectral analyses to be performed by others, confirming the relevance of PAF to workload and performance^56^.

### The manipulation

The final step is determining the way in which the learning task will be manipulated. In the case of 3D-MOT, since the goal is to track the 4 targets amidst the 4 distractors, the manipulation was designed to recall the identity of the targets. During the movement phase of the trial, *target recall* occurred: a subtle red hue (gradual change over 0.5 s to a maximum of 25% saturation) was applied to the four target spheres if non-optimal brain functioning (a slow PAF) over the precuneus was observed.

### Subjects

The research project was approved by the Université de Montréal ethics committee for health research (Comité d’éthique de la recherche en santé; CERES). All recommended ethics procedures and guidelines were followed, and informed consent obtained from all study participants. Four groups (n = 40) took part in this study. All groups were matched for age (mean = 22.89 years, SD = 2.95; range = 19–29 years) and total years of post-secondary education (mean = 2.38 years, SD = 1.01, range = 1 to 5 years). Specific demographic information for each group is detailed in Table 1. All participants were free of any diagnosed cognitive or emotional deficits and psychoactive medication. The first two groups are presented in Parsons et al^40^ as a part of a study investigating the improvements in cognitive function of standard 3D-MOT training. They are the NT group (n = 10) that underwent standard 3D-MOT training, and the CON group (n = 10) that was a non-active control. In addition to those groups, this study added a NT-NFB group (active neurofeedback group; n = 10) and a NT-NFBs group (sham neurofeedback group; n = 10). All subjects were randomly assigned to groups following a block randomization procedure, and all subjects were trained over the same general timeframe (approximately 6 months).

**Table 1:** Group breakdown demographics.

Group	Description	n	Age (years)	SD	YPSE	SD
NT	*Standard learning experimental*	10	23.54	2.56	2.5	0.97
NT-NFB	*NFB-assisted learning experimental*	10	21.90	2.99	2.2	1.03
NT-NFBs	*Sham-assisted learning control*	10	23.10	3.48	2.4	1.17
CON	*Non-active control*	10	23.02	2.78	2.4	0.84

Group composition and demographic information.

YPSE: years of post-secondary education.

The NT, NT-NFB and NT-NFBs groups all performed 10 training sessions of 3D-MOT, with 2 training sessions a week over a period of 5 weeks. Each session lasted approximately 45 min. The CON group was a non-active control and thus underwent no training. All groups also conducted an initial testing session and a final testing session of the standard, un-manipulated 3D-MOT task.

**Figure 2:**
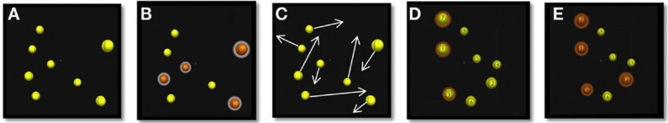
Breakdown of a 3D-MOT trial. (**A**) Presentation: all spheres appear. (**B**) Identification: target spheres are identified in red. (**C**) Movement: spheres move through space following a linear trajectory. (**D**) Response: participant attempts to identify targets. (**E**) Feedback: correct targets are revealed.

The 3D-MOT task took place in a virtual 3D cube with each side measuring 1.5 m and projected onto a square screen with sides measuring 2.4 m. Target and distractor spheres each measured 10 cm in diameter. The speed is measured in meters per second (m/s); each trial began with an initial start speed of 0.3 m/s. Figure 2 presents a breakdown of a 3D-MOT trial.

Participants in the NT-NFB group underwent 3D-MOT training with a manipulation of the task based on real-time EEG-based brain activity. As discussed above, the sole information provided to the subject via neurofeedback-based modulation was the *target recall*. Specifically, the PAF measured over the midline parietal cortex (10–20 electrode site Pz^47^) was used to modulate the targets, making them slightly heterogenous. Pz was chosen as the site for training as it is heavily involved in visuotpatial tasks^46^, as well as proposed as a part of the neurofeedback “control network”, potentially in part responsible for the acquisition of neural autoregulation^57^. Fz was passively recorded for further offline analysis.

Prior to each series of trials, a 1-min baseline was taken, and a PAF threshold was then set as 95% of the baseline level (for example if PAF = 10 Hz, PAF threshold = 9.5 Hz). This measure ensured consideration of the individual and contextual variability inherent in EEG measures. Target recall was based on real-time EEG activity and would occur whenever the PAF dropped below threshold, between seconds 2 and 6 of the movement phase of a trial (see Fig. 2, image C). Target recall was a subtle red hue (gradual change over 0.5 s to a maximum of 25% saturation) applied to the four target spheres. No target recall occurred during the first two and final two seconds of each trial. Further, target recall was discontinued immediately once the PAF returned above threshold, or after a maximum of 0.5 s. Finally, every instance of target recall was followed by 1.5 s of ‘dead time’ during which no additional feedback could be given. Thus, a maximum of 1 total second of feedback time could be given per trial, accounting for 12.5% of the 8-s trial.

The NT-NFBs group was an active control group. The NT-NFBs group underwent the same training as the NT-NFB group, however target recall was not dependent on their brain activity. Instead, while each subject in the NT-NFBs group were told they were receiving feedback based on their brain activity, in reality each subject was paired with a participant in the NT-NFB group, and the color-change they observed was based on the pre-recorded EEG of their pair. The study respected a single-blind design; the experimenters were aware of who received real and sham recall but the participants themselves were not. As such, the task, including amount and timing of target recall was identical for both NT-NFB and NT-NFBs groups, with the only differing variable being that the NT-NFB group received brain-based target recall, while the NT-NFBs group received non-contingent or *sham* target recall.

All subjects were given identical instructions for the task itself incling a brief demonstration of 3D-MOT trial in order to educate them on how to perform this task. NT-NFB and NT-NFBs groups were also told that the target spheres would turn slightly red when an inefficient brain activity was detected.

### Materials

A ProComp Infiniti encoder manufactured by Thought Technology Limited^58^ was used to acquire 2-channel EEG data from active sites Pz & Fz^47^, referenced to linked ears, with the ground at Cz. Application of electrodes was done by first lightly abrading the skin using NuPrep gel, then affixing the electrode to the ear or scalp using Ten20 conductive paste. Impedance for all electrodes was held below 5 kOhm, and within 1 kOhm of one another.

Thought Technology Limited’s BioGraph Infiniti software version 6.0^58^ was used, with a customized interface allowing for communication with the 3D-MOT software program. The EEG signal was sampled at 256 Hz, with a high-pass (0.5 Hz) and low-pass filter (50 Hz). PAF was calculated by isolating the discrete frequency producing dominant power with a 1-s sliding-window FFT of EEG activity between 8 and 13 Hz. Real-time PAF was thus consistently a discrete frequency; either 8, 9, 10, 11, 12 or 13. The running average of PAF over a given trial, series or session could be any decimal value between 8 and 13 Hz.

The 3D-MOT task was custom-coded for the purposes of this project, built from a lab version of the commercially available NeuroTracker (CogniSens Inc.)^59^. The 3D-MOT sessions were performed in the C.A.V.E. (Cave Automatic Virtual Environment)^60^. The C.A.V.E is a 10 foot by 10 foot by 10 foot enclosure onto which is projected the 3D-MOT task. The MOT environment consists of a large cube measuring approximately 1.5 m in length, width, and height, while targets measure 10 cm diameter. The participant is seated at a distance of 1.5 m from the screen and is given a fixation point located in the center of the cube. The 3D-MOT task thus utilises a visual field of approximately 45 degrees. The use of a cube as the environment allows for horizontal and vertical movement of both targets and distractors to remain roughly equivalent. The 3-D aspect of the MOT task is achieved using stereoscopic projection and active shutter lenses synchronized to 120 Hz.

## Results

**Figure 3:**
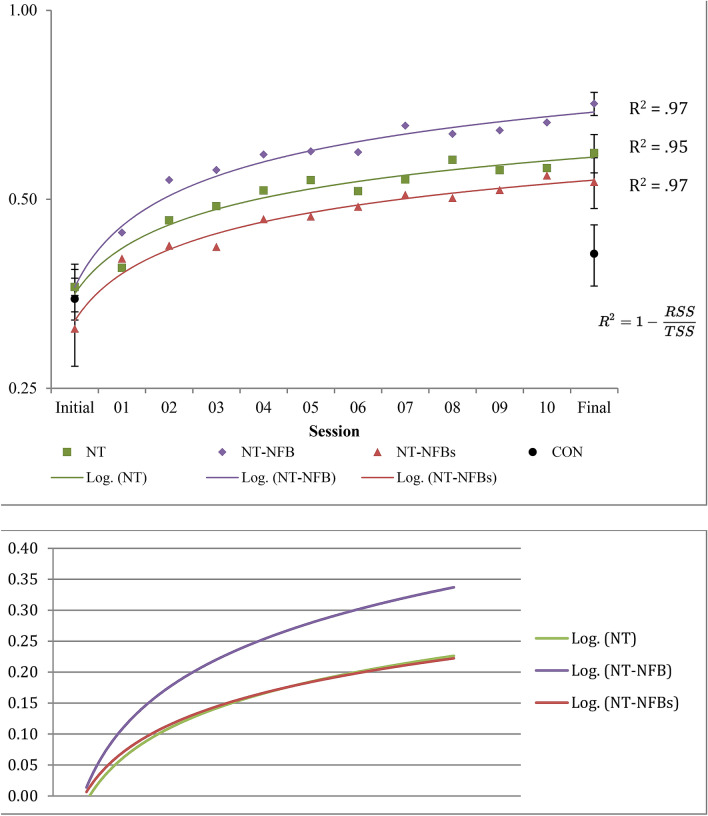
3D-MOT Geometrical Speed Threshold Means on a log scale. Above: Error bars represent standard error and are only presented for Initial and Final sessions in order to maintain image clarity. Standard error for training trials varied between .03 and .05 for the NT group, .02 and .04 for the NT-NFB group, and .04 and .06 for the NT-NFBs group. Lines represent the log regression and the R^2^ corresponds to the amount of variance explained by the fit. (RSS = residual sum of squares, TSS = total sum of squares). Below: Normalized learning curves calculated by taking each individual session score and subtracting the baseline (initial session) score.

The session scores and logarithmic learning curves of each group are presented below in Fig. 3. In the figure, the graph above shows the raw scores while the graph below shows scores based on a normalized baseline (session score—baseline score). A repeated-measures ANOVA of initial and final session scores demonstrated an effect of group (F = 20.317, p =  < 0.001, partial Eta squared = 0.629). As can be easily observed, the NT-NFB group performed better than the NT-NFBs and NT groups, whose results are similar. Finally, as expected, the non-active CON group demonstrated the least amount of learning. In terms of the learning curves, as can clearly be seen in Fig. 3, the NT-NFB group demonstrates a steeper slope and this better learning than all other groups, and this effect is even more evident when observing the normalized learning curves.

In Table 2, the results of a univariate ANOVA on final test scores demonstrate that at final testing, the NT-NFB group significantly outperformed all other groups, while the NT and NT-NFBs groups performed significantly better than the CON group. Table 2 also gives us an idea of the power of the various learning paradigms; the NT-NFB paradigm significantly outperformed all others with effect sizes in the medium to large range. Meanwhile, no significant difference was observed between the classic paradigm (NT) and the sham paradigm (NT-NFBs). Both of these latter paradigms outperformed the non-active control group, with the classic paradigm and sham paradigms presenting a large and medium effect size, respectively. Between-group comparisons of the NT and NT-NFBs are non-significant, and future research could examine this possibility in a larger study.

**Table 2:** Final testing 3D-MOT session threshold score comparison.


Group	Comparator	Significance (p)	Effect size Cohen’s d (r)
NT-NFB	Versus NT	.044	0.9484 (.4284)
	Versus NT-NFBs	.004	1.2742 (.5373)
	Versus CON	< .001	2.3015 (.7548)
NT	Versus NT-NFBs	NS	–
	Versus CON	.003	1.2583 (.5325)
NT-NFBs	Versus CON	.032	0.7317 (.3435)

Y values represent mean speed of the final testing session in metres per second.

Cohen's d = (M2 − M1)/SDpooled (M = mean, SD = standard deviation).

## Discussion

As can be seen from the results section above, the NT-NFB group out-performed all other groups and demonstrated a significantly better learning curve. This could be because the closed-loop learning paradigm provides assistance (target recall) when the learner needs it most, thus keeping the person within the optimal zone of development on a more consistent basis than when using a standard trial-and-error approach (in this case, an adaptive staircase) alone.

This is supported by observations of the NT group: while adaptive staircases based on behavioural performance work and significant learning is observed, a system that integrates real-time cognitive performance into the learning paradigm yields superior results. If the task is simply made easier, such as was the case for the NT-NFBs group who received help at the task (target recall) at times when it wasn’t needed, the learner does not perform any better than with the standard task. It may even be possible that the differences in effect sizes between NT and NT-NFBs groups, which are larger for the former, indicate that non-contingent feedback—irrelevant help—is disruptive to the learning process.

Further, even once target recall was removed from the task at the time of final testing, the NT-NFB group continues to perform at levels well above the other groups. The results of the NT-NFB group did not decrease even though they went back to the classic task and were no longer receiving target recall. This should be interpreted as a confirmation that there is a true acquisition of what was learned. This is an important point: if there were no transfer to classic applications, a closed-loop learning system would be of limited value because a person would always have to find themselves within a closed-loop context to perform at their full potential. This is not the case, as the NT-NFB group demonstrates that their learned abilities transfer back out into a traditional context.

This addresses a significant weakness in existing cognitive enhancement literature^1^ as well as in some classic neurofeedback applications^2^. By integrating a brain-based cognitive enhancement technique into a given learning paradigm, the highly-efficient closed-loop learning paradigm described by Clow^8^ can be achieved, side-stepping the need to achieve more distant transfer.

If we extrapolate, instead of applying neurofeedback in an experimental setting and attempting to achieve transfer, we can imagine designing specific in-classroom neurofeedback protocols for the “live” enhancement of cognitive functions effected by neurodevelopmental disorders such as ADHD^13^, learning disorders^61^, autism spectrum disorders^62^, as well as personal/professional protocols for emotional regulation^16^ and for peak performance^18–20,28–33^. Married with today’s technology, one can even begin to imagine the various forms in which this could take shape: an adaptive augmented reality classroom, the teacher able to modify their lesson based on the real-time brain-states of their student; a book that adapts how it presents information to its reader based on their real-time information processing, comprehension, memory encoding and reasoning; and a virtual reality environment that pushes behavioural and emotional regulation capacities to the maximum, without the risk of ever pushing beyond a tolerable level. Indeed, some of these biofeedback devices exist, most notably capitalizing on heart rate signals in order to manage stress^63^ and for personal emergency response systems worn during exercise^64^.

## Conclusion

This study demonstrates that a closed-loop learning paradigm that incorporates cerebral performance (PAF) as well as cognitive performance (workload) optimizes a person’s ability to learn within the framework of a novel task (3D-MOT). There is still much work to be done before this type of learning system can be implemented on a broader scale.

First and foremost, the results of this study need to be replicated on a larger scale within the context of the 3D-MOT paradigm. Ideally, a larger number of subjects should be trained in order to validate and enhance statistical measures. Moreover, transfer measures such as structural and/or functional brain scans; cognitive performance tests and outcome measures should be utilized to examine the potential for cognitive enhancement beyond the task at hand.

Next, a closed-loop learning paradigm should be tested within the context of other tasks. Other measures of workload could be used, various electrode sites targeted for their involvement in a given task, and the same type of paradigm could theoretically be done using more precise brain-imaging techniques such as real-time functional magnetic resonance imaging (rt-fMRI), yielding deeper insights into task-related brain functioning^65^.

This study should be considered a proof of concept; a demonstration that it is possible to use brain activity to improve how the brain learns, by consistently presenting it with information within the zone of proximal development. While the immediate impact of this one study may be limited, there are far reaching implications: the possible real-world applications of a closed-loop learning paradigm are considerable, research supports PAF as a task-independent measure appropriate to multiple paradigms^52^, and the potential gains in learning outcomes across a wide variety of domains and applications are immense.

## Data Availability

The datasets generated and analysed during the current study are available from the corresponding author on reasonable request.
